# Supplementary material for: Chromatin Accessibility and Transcriptional Landscape during Inhibition of Salmonella enterica by Lactobacillus reuteri in IPEC-J2 Cells
Source: Cells. 2023 Mar 22;12(6):968. doi: 10.3390/cells12060968 (PMC10046971; doi:10.3390/cells12060968)
Supplement: Supplementary file 1 [file cells-12-00968-s001.zip › Table S1.pdf]

Table S1. Details of primers and antibodies

GENE	Sequence (5’→3’)	Product length (bp)
HILA	F: 5'-TCCACGCAGGAAATAACAGG-3'<br>R: 5'-TGGGCAACCAGCACTAACG-3'	203
INV1	F: 5'- GGCGATCCTTGAACAAATAGC -3'<br>R: 5'- CGGCGAACAATAGACTGCTT -3'	145
16S	F: 5'- GGCGATCCTTGAACAAATAGC -3'<br>R: 5'- CGGCGAACAATAGACTGCTT -3'	180
UBTD2	F: 5'-TTGTGCCGCCTTGAGATA-3'<br>R: 5'-TTGCAGGAAGAATACTGATTTA-3'	286
LRRC39	F: 5'- CTATGCCTCAGTGACGTG-3'<br>R: 5'- TCTGCCAAAGTGGTTATG -3'	248
GRIK4	F: 5'- AGTGCTGCTATGAATGGT-3'<br>R: 5'- GTGTTGGCAAAGATGTGG-3'	198
ENSSSCG00000014143	F: 5'- CCAGCACCTAAACGACTT-3'<br>R: 5'- ATGCTTTGGTTTTATGGC-3'	277
ARNTL2	F: 5'- ACTGGGCCGTATGATAGT -3'<br>R: 5'- AGATGCTGGAAGGGTGTA -3'	132
PIK3R1	F: 5'- AGCCAAGGAAACTCTCGCA -3'<br>R: 5'- CATTCACTTCTTCCCTCGAGAT -3'	245
**Antibodies**	**Source**	**Application**
PI3KR1	HUABIO	WB
P-AKT	Abcam	WB
AKT	Abcam	WB
P-JNK	Abcam	WB
JNK	Abcam	WB
P-ERK	Abcam	WB
ERK	Abcam	WB
Cyclin D1	HUABIO	WB
CDK4	HUABIO	WB
BCL-2	HUABIO	WB
BAX	HUABIO	WB
HSP90	HUABIO	WB
