# Supplementary figures and images for: Chromatin Accessibility and Transcriptional Landscape during Inhibition of Salmonella enterica by Lactobacillus reuteri in IPEC-J2 Cells
Source: Cells. 2023 Mar 22;12(6):968. doi: 10.3390/cells12060968 (PMC10046971; doi:10.3390/cells12060968)

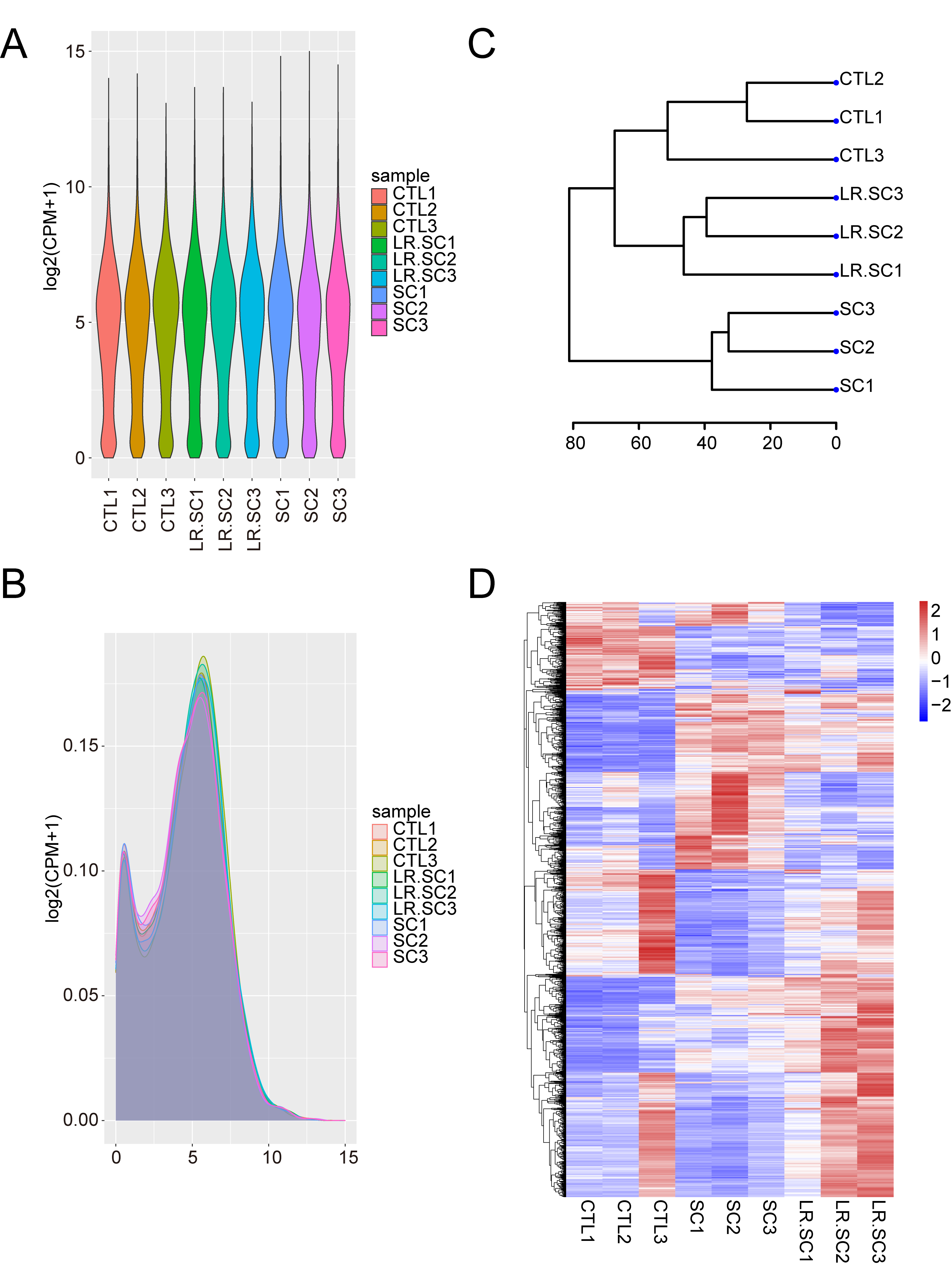

Supplement: Supplementary file 1 [file cells-12-00968-s001.zip › Figure S1.tif]

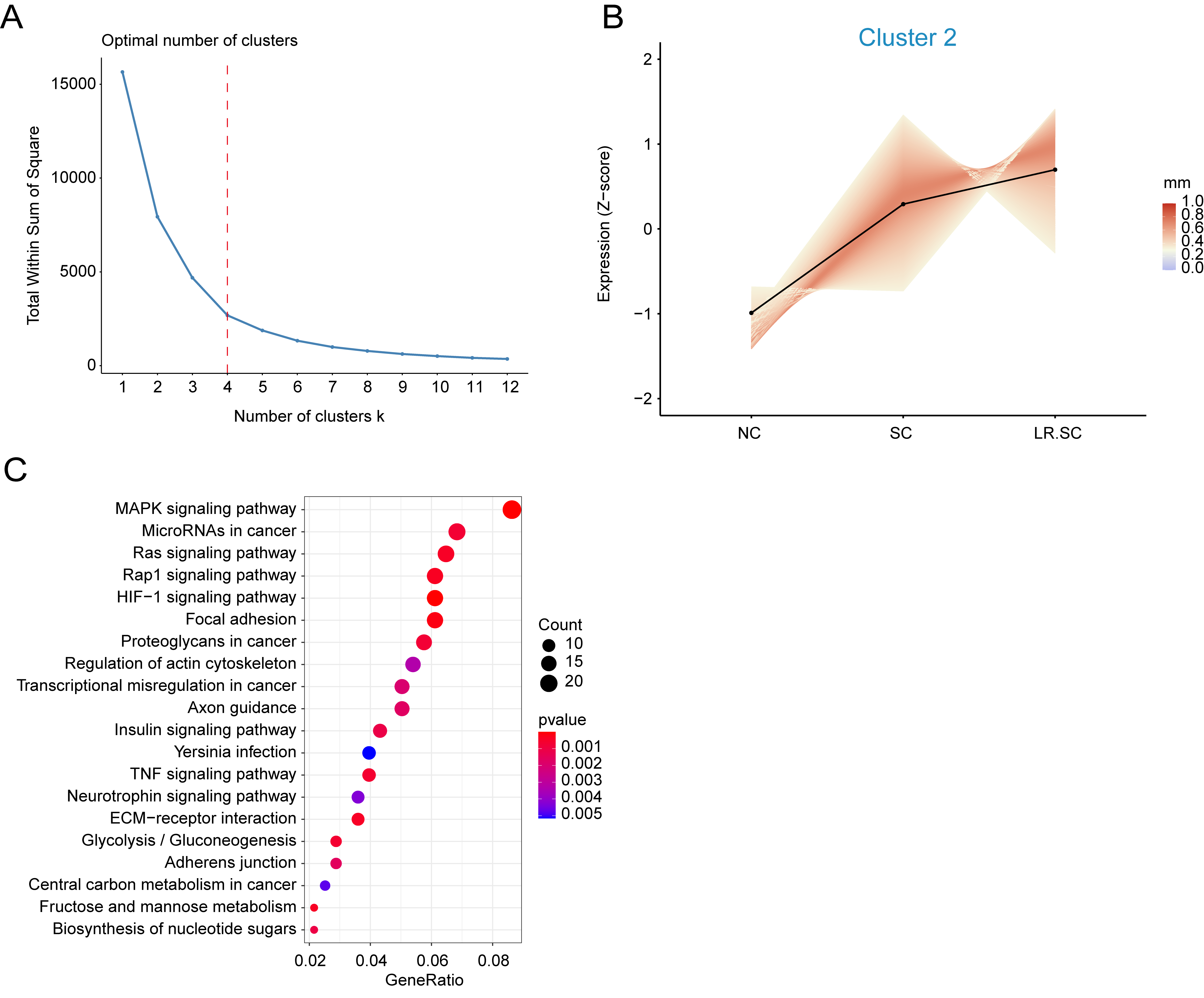

Supplement: Supplementary file 1 [file cells-12-00968-s001.zip › Figure S2.tif]

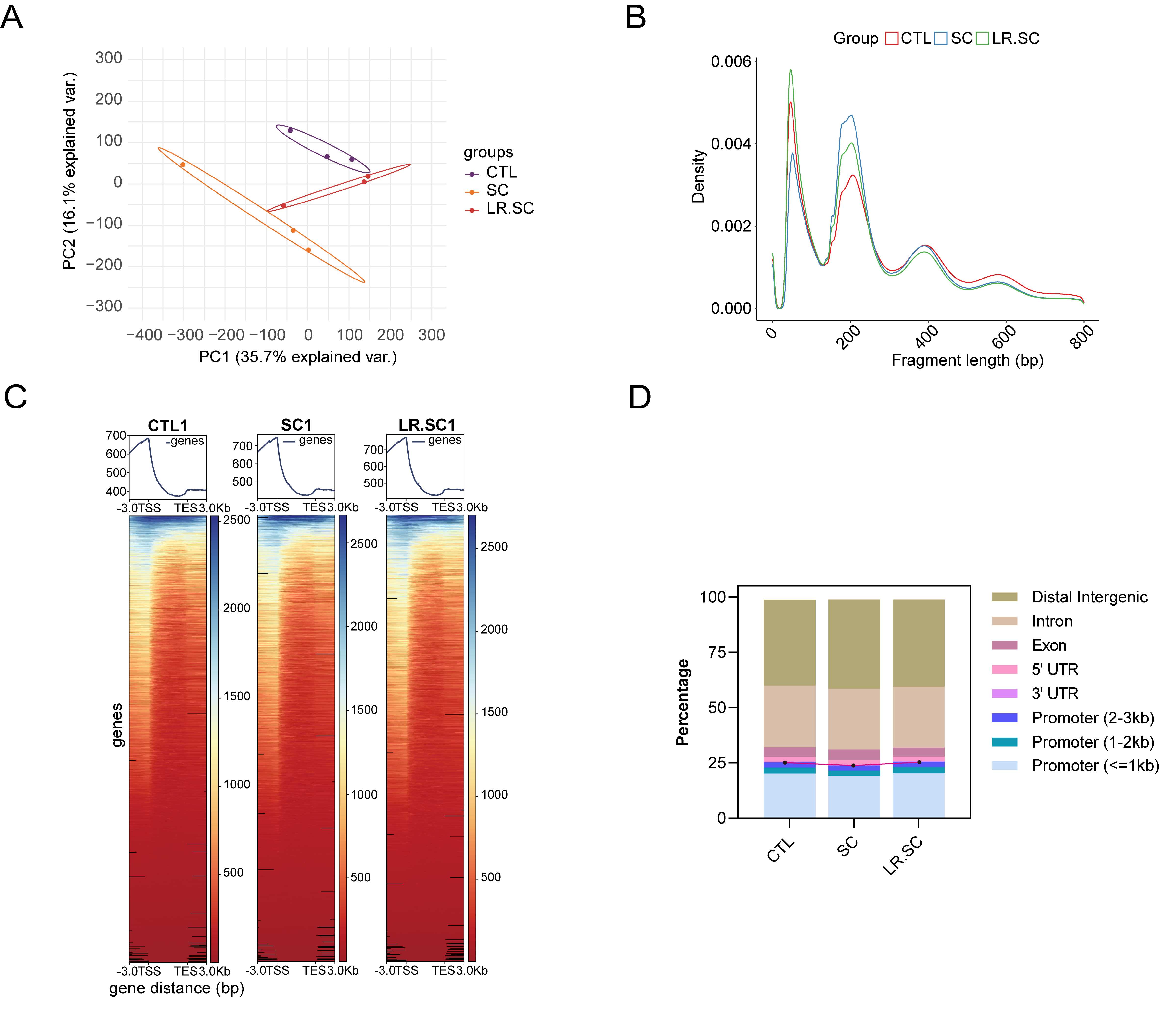

Supplement: Supplementary file 1 [file cells-12-00968-s001.zip › Figure S3.tif]

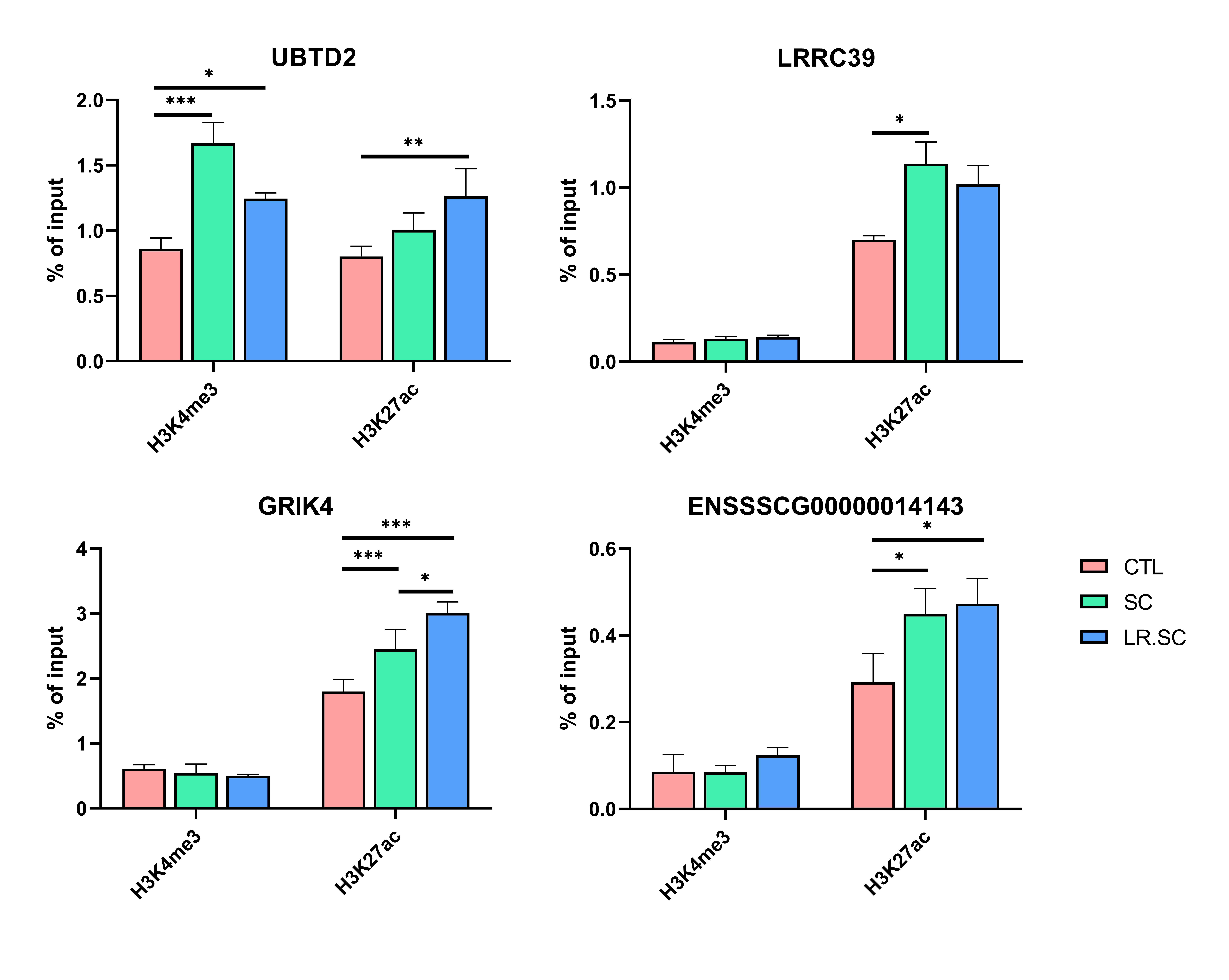

Supplement: Supplementary file 1 [file cells-12-00968-s001.zip › Figure S4.tif]

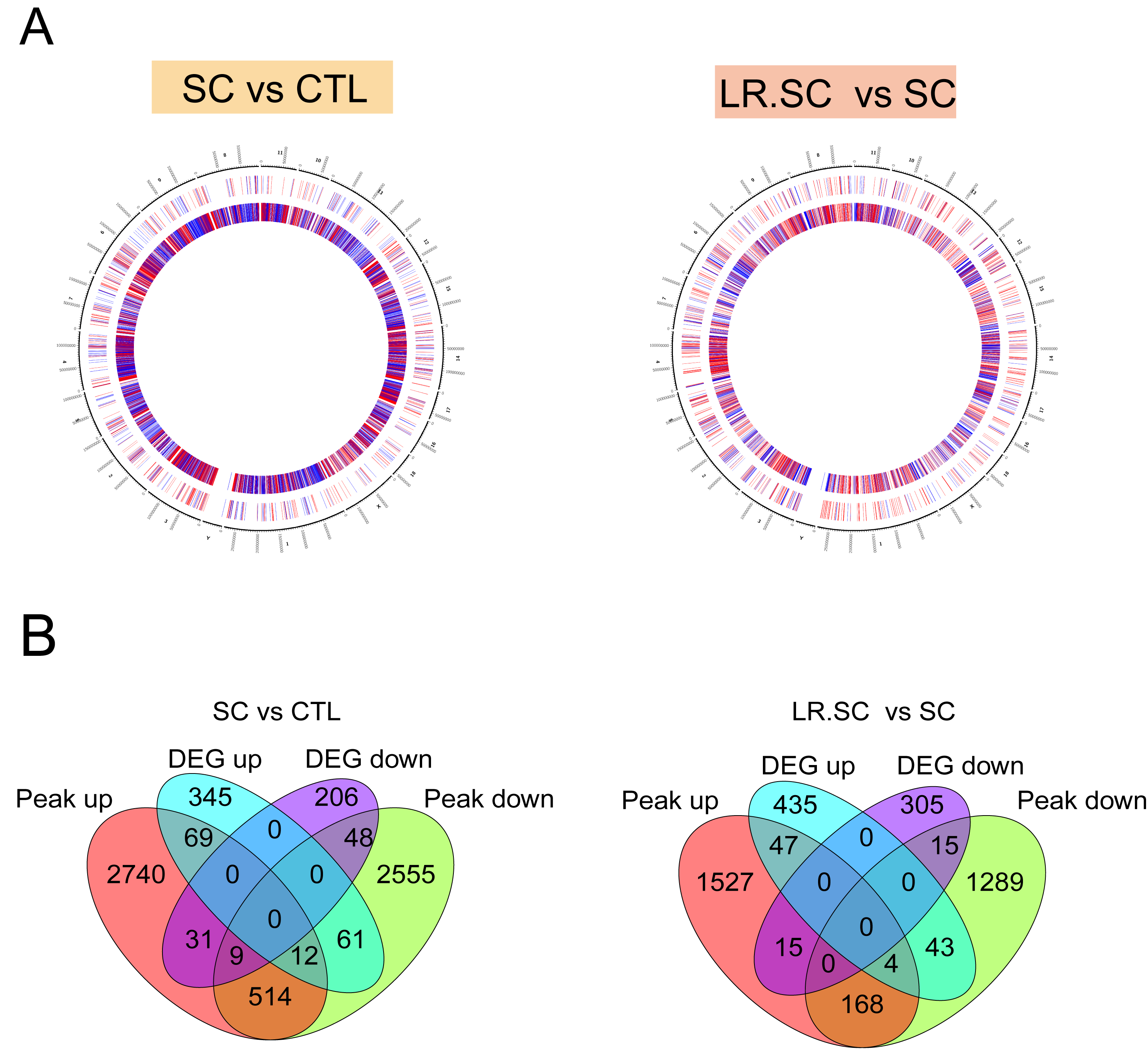

Supplement: Supplementary file 1 [file cells-12-00968-s001.zip › Figure S5.tif]
